# Cysteine Cathepsins as Regulators of the Cytotoxicity of NK and T Cells

**DOI:** 10.3389/fimmu.2014.00616

**Published:** 2014-12-02

**Authors:** Milica Perišić Nanut, Jerica Sabotič, Anahid Jewett, Janko Kos

**Affiliations:** ^1^Department of Biotechnology, Jožef Stefan Institute, Ljubljana, Slovenia; ^2^Division of Oral Biology and Medicine, The Jane and Jerry Weintraub Center for Reconstructive Biotechnology, UCLA School of Dentistry, University of California Los Angeles, Los Angeles, CA, USA; ^3^Faculty of Pharmacy, University of Ljubljana, Ljubljana, Slovenia

**Keywords:** cathepsins, cystatins, cytotoxicity, natural killer cells, cytotoxic T cells

## Abstract

Cysteine cathepsins are lysosomal peptidases involved at different levels in the processes of the innate and adaptive immune responses. Some, such as cathepsins B, L, and H are expressed constitutively in most immune cells. In cells of innate immunity they play a role in cell adhesion and phagocytosis. Other cysteine cathepsins are expressed more specifically. Cathepsin X promotes dendritic cell maturation, adhesion of macrophages, and migration of T cells. Cathepsin S is implicated in major histocompatibility complex class II antigen presentation, whereas cathepsin C, expressed in cytotoxic T lymphocytes and natural killer (NK) cells, is involved in processing pro-granzymes into proteolytically active forms, which trigger cell death in their target cells. The activity of cysteine cathepsins is controlled by endogenous cystatins, cysteine protease inhibitors. Of these, cystatin F is the only cystatin that is localized in endosomal/lysosomal vesicles. After proteolytic removal of its N-terminal peptide, cystatin F becomes a potent inhibitor of cathepsin C with the potential to regulate pro-granzyme processing and cell cytotoxicity. This review is focused on the role of cysteine cathepsins and their inhibitors in the molecular mechanisms leading to the cytotoxic activity of T lymphocytes and NK cells in order to address new possibilities for regulation of their function in pathological processes.

## Introduction

Peptidases are enzymes that catalyze the hydrolysis of peptide bonds in a polypeptide chain. On the basis of their catalytic mechanism they can be divided into seven main groups: cysteine, serine, aspartyl, asparagine, glutamic, threonine, and metallo peptidases (1). Exopeptidases remove residues from N- or C-terminal end of the polypeptide chain, whereas endopeptidases catalyze the cleavage or hydrolysis of peptide bonds within the polypeptide chain. Furthermore, peptidases can act degradatively, causing complete breakdown of the targeted protein substrate, or regulatorily, cleaving one or more specific bonds, thus, altering the biological function of the target. In biological systems, the proteolytic activity of peptidases is controlled by a variety of mechanisms: (1) regulation of their expression at transcriptional and/or translational levels; (2) synthesis of peptidases as inactive zymogens; (3) activation of peptidases by co-factors; (4) recognition and cleavage of a particular peptide bond flanked by specific amino acids; (5) compartmentalization in granules or endolysosomal vesicles thus separating them from their substrates; and (6) binding of peptidase inhibitors to the active site preventing access to substrates (2). In this review, only the latter, i.e., the control of their activity by protease inhibitors is described in more detail.

Peptidases are involved in many biological processes, the most studied being protein catabolism and apoptosis as well as their role in inflammation, regulation of hormone processing, bone resorption, and blood clotting. In immune processes, the peptidases control key functions of innate and adaptive immune responses such as antigen processing and presentation to T cells, host–pathogen cross-talk, cell cytotoxicity, and resistance to bacterial and viral infections (3). Their dysregulated function has been associated with various pathological processes including processes related to altered function of the immune response, such as inflammation, allergies, autoimmune disorders, and impaired anti-tumor immune response (4–6). The role of cysteine cathepsins in regulating the cytotoxicity of cytotoxic T lymphocytes (CTLs) and natural killer (NK) cells is particularly addressed in this review in order to highlight the recent developments in this field and to emphasize the importance of these peptidases and their endogenous and exogenous inhibitors for normal functioning of important effector cells of the cellular immune response.

Of the numerous peptidases involved in physiological and pathological processes of the immune response, a group of endolysosomal cysteine peptidases, also named cysteine cathepsins, attracted a lot of attention during last years. The best known roles of cysteine cathepsins in these processes are their contributions to major histocompatibility complex (MHC) class II antigen presentation, cytokine and growth factor degradation, promotion of adhesion and migration of immune cells and regulation of cell cytotoxicity by processing pro-granzymes into proteolytically active forms (5, 7–9). The role of cysteine cathepsins in regulating the cytotoxicity of CTLs and NK cells is particularly addressed in this review in order to highlight the recent developments in this field and to emphasize the importance of these peptidases and their endogenous and exogenous inhibitors for normal functioning of important effector cells of the cellular immune response.

## Cysteine Cathepsins

In the human genome, there are 561 genes encoding peptidases. Of these, 148 encode cysteine peptidases, among them a group of 11 lysosomal cysteine peptidases, the cathepsins (10). Cysteine cathepsins exhibit different level of expression and specificities, all of which contribute to their different physiological functions. Some of cathepsins, like B, H, L, and C, are ubiquitous in cells and tissues, whereas others, such as cathepsins S, X, V, O, F, K, and W can be found only in specific cell types.

The majority of cysteine cathepsins act as endopeptidases, the exopeptidases are cathepsins B, C, X, and H (1, 11). The exopeptidases possess additional structural elements in the vicinity of their active sites that prevent binding of extended natural substrates into the active site cleft and providing additional segments to facilitate binding of the C- or N-terminal end of the substrate (12, 13). Owing to these structural differences cathepsins B (also known as cathepsin B1 and longipain) and X (also known as cathepsin, Z, P, IV/B2/Y and lysosomal carboxypeptidase B) can act by cleaving their substrates as a dipeptidylcarboxypeptidase and a carboxymonopeptidase, respectively (14, 15) while cathepsins C (also known as cathepsin J and dipeptidyl peptidase I – DPPI) and H (also known as cathepsin B3/I) cleave their substrates as aminopeptidases (1, 16). Cathepsins B and H may retain their endopeptidase activity, in addition to their exopeptidase activity, depending on the local pH (17, 18). Cysteine cathepsins were classified as cathepsin L-like and cathepsin B-like enzymes on the basis of the ERFNIN motif that is conserved in the propeptide of cathepsin L-like enzymes, but not cathepsin B-like enzymes (19). However, considering the structural and genetic data available in recent years, they can be divided into at least four groups (B-, L-, X-, and C-like cathepsins) (20).

Cysteine cathepsins were long viewed as enzymes involved in final protein degradation in the lysosomes. More recently, they have been associated with more specific functions in a variety of important physiological and pathological processes (21). Cysteine cathepsins can be implicated also in processes occurring outside lysosomes, e.g., in the nucleus, on the cell membrane, the cytosol, and they can even be secreted into the extracellular environment (3).

## Endogenous and Exogenous Inhibitors of Cysteine Cathepsins

The activity of cysteine cathepsins is ultimately controlled by endogenous peptidase inhibitors, such as cystatins, thyropins, propeptides, members of the serpin (serine peptidase inhibitors) family, and by the general peptidase inhibitor α2-macroglobulin (22). Among them the most important are cystatins that comprise a superfamily of evolutionarily related proteins present in all living organisms, and also in viruses. Type I cystatins (also known as stefins) are cytosolic and nuclear proteins. They are 100 amino acid long, single chain proteins, they do not contain disulfide bonds and are not glycosylated (23). In contrast, type II cystatins are typical extracellular proteins, 120 amino acid long, possessing two disulfide bonds (24). The members of family II are cystatins C, E/M, F, D, S, SA, and SN, and male reproductive tract cystatins 8, 9, 11, 12, CRES2, CRES3, cystatin 13, and cystatin 14 (1). Kininogens, large, multifunctional plasma proteins, represent type III cystatins. They contain three type II cystatin-like domains and they are precursors of the vasoactive peptide kinin (25). Fetuins and latexins represent additional protein families and comprise two cystatin-like domains; however, they do not exhibit inhibitory activity against cysteine peptidases (23). The tertiary structure of cystatins is conserved and exhibits the typical cystatin fold (26). The N-terminal segment and two cystatin loops form a wedge-shaped edge that is complementary to the active site cleft of the peptidase. Cystatins are tight-binding inhibitors of the C1 family of cysteine peptidases, while some type II cystatins (C, E/M, and F) also have a second reactive site for inhibiting members of the C13 family of cysteine peptidases (27). Physiologically, cystatins regulate excessive cysteine proteinase activity in cells, tissues, and body fluids. In general, they act as “emergency” inhibitors, trapping and neutralizing peptidase activity. However, cystatins are present in cytoplasm and in extracellular fluids whereas their targets, cysteine cathepsins, are localized predominantly in lysosomes. Therefore, it is not clear under what circumstances cystatins meet their targets. In recent studies, it was shown that secreted type II cystatins can be internalized by immune or tumor cells, accumulating in endosomal/lysosomal vesicles (28, 29). The vesicular localization of cystatins may result in affecting a number of cell functions.

The number of exogenous inhibitors capable of inhibiting cysteine cathepsins is increasing and comprises protein and non-protein molecules isolated from animals, microorganisms, plants, and fungi, neutralizing monoclonal antibodies and small synthetic molecules. However, only a few of them have the potential to be applied in clinical studies for treating diseases associated with an excessive activity of cysteine cathepsins. Among the inhibitors of animal, plant, and microbial origin the best known cysteine peptidase inhibitor is E-64 (1-[l-*N*-(trans-epoxysuccinyl)leucyl] amino-4-guanidinobutane), isolated from *Aspergillus japonicus* (30). Its epoxysuccinyl scaffold has served for the synthesis of a series of derivatives with improved specificity toward particular peptidases (31). Owing to their poor cell permeability, off-target binding, and the irreversible nature of their inhibition, the epoxysuccinyl inhibitors have not been introduced into clinical practice. The development of synthetic peptidase inhibitors has been directed toward reversible inhibitors that bind peptidases either covalently or non-covalently (32). Some of them have proved promising at the preclinical level and are therefore good candidates to enter clinical testing in the near future.

## Cytotoxic Cells

Cytotoxic T lymphocytes and NK cells are the major components in the effector arm of the cellular immune response against pathogen infected and tumor cells. Despite many fundamental biological differences between them, both CTLs and NK cells employ the same mechanism to initiate target cell apoptosis. One involves the death receptor pathway, encompassing Fas (CD95)-Fas ligand (CD95L) and related death receptor mediated pathways (33). The other is the granule exocytosis pathway, and involves regulated release of the contents of cytotoxic granules into the immunological synapse formed between the effector and target cell (33). The granule exocytosis pathway is the principal killing mechanism employed by CTLs and NK cells in combating numerous viral infections (33–35) as well as some tumors (36, 37). It is also an important pathogenic mechanism underlying organ allograft rejection and graft vs. host disease (35, 38).

The key components that induce cell death in target cells are contained in cytotoxic granules, complex organelles that combine specialized storage and secretory functions with general degradative functions of typical lysosomes (39, 40). The most prominent components of this “death cargo” are perforin and a family of serine proteases, termed granzymes (39). In addition, cytotoxic granules contain several important lysosomal hydrolases including peptidases cysteine cathepsins C, H, and L, as well as lysosomal membrane proteins Lamp-1, Lamp-2, and Lamp-3 (41, 42).

*Perforin* is a calcium-dependent, pore-forming member of the membrane attack complex/PRF (MACPF) protein family (43, 44). It is synthetized as an inactive precursor, which requires the removal of 20 aminoacids of the C-terminus for its activation (45). This processing event is believed to unmask the perforin C2 domain, enabling it to bind to the cell membrane (45). Perforin is essential for the entry of granzymes into the cells and *in vitro* experiments have shown that loss of perforin leads to complete failure of effector cells to lyse targets (46, 47). Furthermore, perforin-deficient mice are susceptible to numerous immunogenic challenges that are normally eliminated by cytotoxic cells (46, 48). In human beings, perforin deficiency has been shown to be the underlying mechanism in the development of familial hemophagocytic lymphohistiocytosis (HLH) pathogenesis, since 30–40% of such patients have mutations in both copies of the perforin gene, leading to complete loss of function (49). As a consequence, they develop severe immunoregulatory disorder in infancy (49).

*Granzymes* belong to a family of neutral serine proteases. Seven different granzymes have been identified in the mouse (granzymes A–G) (50, 51) and five in human beings (granzymes A, B, H, M, and K) (52–54). All five human granzymes have been shown to initiate caspase-dependent or independent cell death (55). The most abundant and the most studied are granzymes A and B (33). Granzymes H and M are preferentially expressed by NK cells (52, 56), while granzyme K is more restricted to lymphocytes of the T cell lineage (53, 54). All granzymes are initially synthesized as zymogens, being finally converted into active enzymes inside secretory granules by cleavage of the dipeptide (usually Gly–Glu or Glu–Glu) from their N terminus (57). The granzymes differ in their substrate specificity and cleave distinct target cell proteins to initiate apoptosis (58–60). While complete loss of exocytosis-dependent cytotoxicity is documented in perforin-deficient mice, a milder deficiency has been observed in individual granzyme gene-knockout animals, suggesting that a redundancy of function between individual granzymes with regard to viral infections and tumor control (61–64).

Other, non-cytotoxic roles of granzymes have recently been detected. Granzyme A has been shown to promote release of proinflamatory cytokines, such as IL-1β from primary mouse macrophages (65) while granzyme B can cleave many extracellular matrix (ECM) components, thus, promoting detachment-induced cell death, or anoikis (66). In addition, granzyme B is involved in regulating the function and maintenance of T helper cell populations (67), and the direct cleavage of viral proteins (68).

### Natural killer cells

Natural killer cells constitute only 10% of lymphocytes. However, they are major cytotoxic effectors of innate immune responses toward pathogens, most notably viruses (69) and transformed and senescent cells (70, 71). In human beings, NK cells develop from CD34^+^ hematopoietic cells in the bone marrow and undergo terminal maturation in secondary lymphoid tissues (72). On the basis of surface CD56 expression, two major subsets of NK cells can be distinguished in human peripheral blood, namely, CD56^dim^ and CD56^bright^ NK cells (72). CD56^dim^ NK cells are the predominant mediators of the cytotoxicity response. They are fully mature, and constitute 90% of the NK cells in peripheral blood, while CD56^bright^ cells are immature-like, and have been considered primarily as cytokine producers (73).

Natural killer cells are independent of antigen presentation (74). Their activation is inhibited through the interaction of their inhibitory receptors with MHC class I molecules (75), which are expressed on nearly every healthy cell of the body (76). Although it was initially thought that NK cell activation is triggered only by the lack of interaction with MHC class I molecules (76), further studies have revealed that it is a more complex process, involving a number of structurally distinct germline-encoded receptors (77). Activation receptors can be grouped, according to their signaling pathways, in three categories: those that signal through immunoreceptor tyrosine-based activation motif (ITAM)-containing subunits (e.g., CD16, NKp46, NKp44), through the DAP10-associated receptor NKG2D, and through other receptors (e.g., CD2, 2B4, DNAM-1) that signal by different pathways. These receptors usually synergize to activate NK cells, so the activation in their recognition of a target is achieved after a favorable balance of activation over inhibitory signals (78, 79).

Since NK cells exist in a “pre-activated” state (74) and are capable of secreting cytotoxic granules almost immediately upon activation (80), multiple checkpoints are in place to avoid inappropriate degranulation and damage to healthy cells (81, 82). In addition, the granule polarization and degranulation arising from NK cell activation are under the control of different activation pathways [reviewed in Ref. (79)].

Apart from their cytolytic function, NK cells contribute significantly to the cytokine milieu, and their release upon activation can significantly modulate the subsequent adaptive immune response. More precisely, it has been shown that NK cells can stimulate T cell responses to a variety of antigens, by cytokine and chemokine production directly or indirectly, by acting on antigen presenting cells (APCs) (83, 84). Furthermore, in interaction with stem cells and some tumor cells, NK cells almost completely lose their ability to mediate cytotoxicity but secrete significant amounts of cytokines – a condition termed split anergy (85). NK cells with split anergy may be involved in regulating the differentiation and resistance of a variety of stem cells, including cancer stem cells (86). Furthermore, NK cells can kill activated T cells and APCs directly (87–89). Their ability to dampen an immune response may provide protection in the context of autoimmunity (87, 90).

In addition, new evidence indicates that NK cells can be “educated” and selected during development and that they exhibit antigen specificity and undergoes clonal expansion during infection (91, 92). On a mouse model of cytomegalovirus (CMV) infection, long-lived NK cells have been shown to arise under certain circumstances (91). Furthermore, a population of long-lived NK cells exhibiting enhanced responsiveness to secondary CMV infection was detected in certain human transplant recipients (93, 94). All these newly gained attributes, usually assigned to the components of the adaptive immune system, give rise to a new dimension in the role of NK cells in the immune response.

### Cytotoxic T cells

In general, initial recognition of pathogen infected or transformed cells by the components of innate immunity is followed by the adaptive immune response that is mediated by clonally selected and expanded antigen-specific CTLs.

Cytotoxic T lymphocytes develop in the thymus from a common lymphoid progenitor, through a series of distinct developmental stages that lead to the generation of fully competent, self-MHC I complex restricted, naive CD8^+^ T cells (95). Naive CD8^+^ T cells are small round cells that do not contain cytotoxic granules (96–98). For their full development into effector cells capable of cytotoxic killing, CTLs require appropriate antigen stimulation. The T cell receptor (TCR) serves as the antigen receptor of CD8^+^ T cells. It recognizes an antigenic peptide presented within the antigen-binding groove of a particular MHC class I molecule itself presented on the surface of professional APC, namely, dendritic cells (99). In order to become fully activated and undergo clonal expansion, CTLs cells need to receive a co-stimulatory signal provided by interactions of T cell-specific CD28 molecules with their APC-expressed glycoprotein ligands B7.1 (CD80) and B7.2 (CD86) (100). Appropriate activation triggers synthesis of the lytic proteins in CTLs (98), cell division, and the appearance of fully differentiated, electron-dense cytotoxic granules in the cytoplasm (96, 97). Further recognition of target cells via the TCR on these activated cells triggers polarized movement of cytotoxic granules to the specialized area called the cytolytic synapse and to exocytosis of their contents into the extracellular space (101). This process also reinitiates the cycle of protein synthesis and organelle biogenesis, allowing further rounds of killing to take place (102).

It is important to emphasize that, apart from CD8^+^ T lymphocytes, human CD4^+^ T regulatory cells (Tregs) have recently also been shown to express components of cytotoxic machinery and to kill autologous and allogeneic target cells, including activated CD4^+^ and CD8^+^ T cells, B-cells, monocytes, and both immature and mature dendritic cells, in a perforin- and/or granzyme-dependent manner (103–105). These data emphasize the importance of the cytotoxic death pathway mediated by perforin and granzymes, not only for effective clearance of infections and immune surveillance but also for down regulation of the immune response, maintenance of lymphocyte homeostasis, and prevention of autoimmune diseases.

## Cysteine Cathepsins in the Immune Response

Cysteine cathepsins can be divided into those constitutively expressed in cells and tissues, and those found predominantly in specific cell types. Constitutively expressed cathepsins, such as cathepsins B, L, and H, are present in most immune cells, the highest levels being found in macrophages (106). Specific expression of cathepsin C has been observed in CTLs, macrophages, and granulocytes and mast cells (107, 108), of cathepsin X in monocytes, macrophages, dendritic cells, and T cells (32), of cathepsin S in APCs such as dendritic cells, B-cells, and macrophages (109), of cathepsin F in macrophages (7), and of cathepsin W (also known as lymphopain) in NK cells (110).

The most investigated role of cysteine cathepsins in the immune response is the regulation of MHC class II-dependent antigen presentation (5). This process requires the participation of cysteine cathepsins in two convergent processes: degradation of antigen into antigenic peptides and stepwise degradation of invariant chain (Ii), which blocks MHC class II molecule peptide binding site, to class II-associated invariant chain peptide (CLIP). Cathepsins S and L have been suggested as the key enzymes involved in the processing of Ii. Cathepsin S appears to be essential for removal of Ii in B-cells and dendritic cells, whereas this function is carried out by cathepsin L in thymic epithelial cells (111). In macrophages, both enzymes are present (111). Accordingly, the absence of cathepsins S and L has major consequences for the onset of the T cell dependent immune response. When cathepsin S is missing, the processing of Ii is stopped at p10 fragment and the transport of MHC class II to the cell surface is delayed by its strong retention in lysosomes (112). Similarly, cathepsin L-deficient mice exhibit abnormalities in positive thymic selection due to the accumulation of p10 Ii fragment and defective MHC class II peptide loading in thymic epithelial cells (106, 112). Cathepsin F is another cysteine cathepsin involved in Ii processing in macrophages (7). The different pH profiles of cathepsins F and S suggest that the possibility that they may function in distinct antigen processing compartments. The pH optimum of cathepsin F favors lysosomes, whereas cathepsin S activity is present throughout the endosomal/lysosomal pathway.

Cathepsin X expression is restricted to cells of the immune system, predominantly monocytes, macrophages, microglia, and dendritic cells (113, 114). It acts solely as an exopeptidase and regulates the proliferation, maturation, migration, and adhesion of immune cells, as well as their phagocytosis and signal transduction (8), and therefore, could indirectly affect the function of cytotoxic cells. Recently, several molecular targets of cathepsin X exopeptidase activity have been identified, including integrin receptors, γ-enolase, chemokine CXCL-12, bradykinin, kallidin, huntingtin, and profilin 1 [reviewed in Ref. (32)]. Of these, the interactions with integrin receptors appear to be the most relevant for cathepsin X function in immune cells. Active cathepsin X cleaves sequentially four C-terminal amino acids of the β2 subunit of the β2 integrin receptors that are present predominantly in cells of monocyte/macrophage lineage (115). In monocytes and macrophages, this cleavage results in activation of the β2 integrin receptor Mac-1 (CD11b/CD18) that is associated with increased cell adhesion, phagocytosis, and T lymphocyte activation. In addition, the Mac-1 receptor is crucial for the functioning of dendritic cells, and its recruitment accompanies the adhesion of immature dendritic cells to the ECM. During maturation, cathepsin X translocates to the plasma membrane of maturing dendritic cells, enabling Mac-1 activation and, consequently, cell adhesion. In mature dendritic cells, cathepsin X is redistributed from the plasma membrane back to the perinuclear region, which coincides with the detachment of dendritic cells and acquisition of the mature phenotype (116). In addition to active cathepsin X, its pro-form may also iteract with integrin receptors through RGD (Arg–Gly–Asp) motifs present in its propeptide (117, 118). By binding to integrin receptors such as αvβ3 and αvβ5 that bind RGD, cathepsin X significantly changes cell adhesion to the proteins of ECM (119).

**Figure 1:**
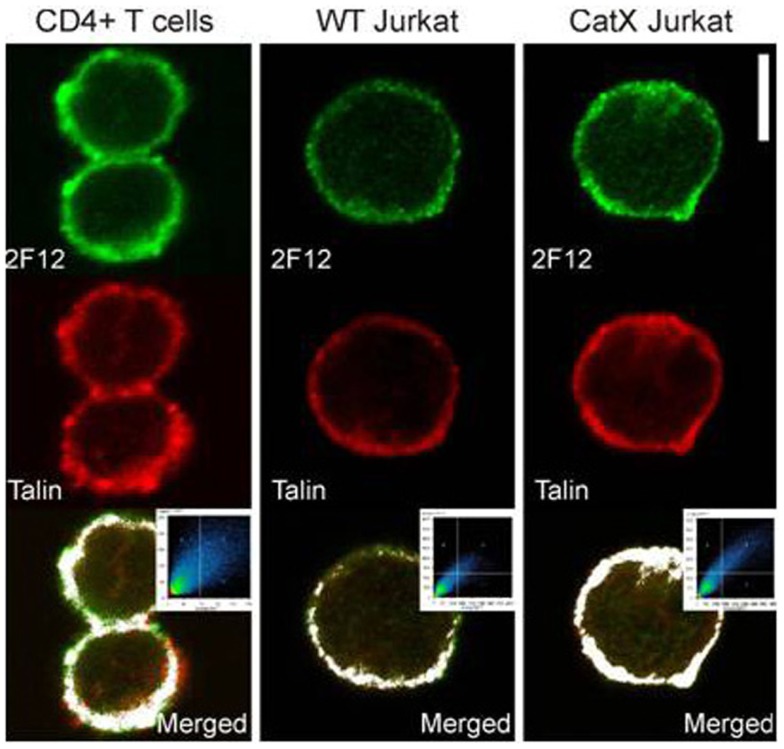
**Colocalization of cathepsin X and adaptor protein talin-1 in T cells (120)**. Primary CD4^+^ T cells, Jurkat cells, and cathepsin X-overexpressing Jurkat cells were allowed to migrate on ICAM-1- precoated slides for 30 min, and analyzed for colocalization of talin and cathepsin X. Specific antibodies for talin-1 (red) and cathepsin X (2F12, green) have been applied. The threshold level for this display corresponds to one-third of the maximal brightness level. Colocalization is represented by the pixels above the threshold in both channels on the contour plot and on the merged image (white color). Original scale bar, 5 μm.

Besides in macrophage/dendritic cell lineages, cathepsin X is present also in T cells (120), although in lower levels (Figure 1). In T cells, cathepsin X regulates the function of another β2 integrin receptor–lymphocyte function associated antigen-1 (LFA-1). LFA-1 is one of the key regulators of physiological T cell functions, including migration and formation of the immunological synapse. The interaction of cathepsin X with LFA-1 promotes cytoskeleton-dependent morphological changes and migration. Gradual cleavage of the β2 cytoplasmic tail of LFA-1 modulates its affinity for the structural adaptors talin-1 and α-actinin-1, enabling a stepwise transition between intermediate and high-affinity conformations of LFA-1 (121). Conformational changes are of vital importance for the regulation of LFA-1 affinity and the binding of ICAM-1. LFA-1 fine-tuning by cathepsin X enables the trafficking of T cells, which is accompanied by extensive actin remodeling via selective binding of actin by talin-1 and α-actinin-1.

Cathepsin X and other cysteine cathepsins (B, K, and S) are expressed also in microglia (122). It is now clear that activation of microglia contributes to neurodegeneration by releasing neurotrophic factors. These act as neuroprotective, proinflammatory, and/or cytotoxic factors that contribute to the degeneration of neurons (123). Cathepsin X secreted from microglia may affect γ enolase, one of the neurotrophic factors present in neurons, by proteolytic cleavage of its C-terminus (9).

Cathepsin C is expressed mainly in cytotoxic cells, mast cells, and neutrophils. Its predominant localization in the secretory granule compartment of these cells is suggestive of its specific function. Thus, cathepsin C has been shown to be indispensable for processing neutrophil serine proteases (granzymes, cathepsin G, and elastase) (124). Accordingly, patients suffering from Papillon–Lefèvre syndrome, characterized by congenital cathepsin C deficiency, exhibit manifestations of neutrophil dysfunction, such as severe gingivitis (125, 126). Furthermore, cathepsin C has been shown to be necessary for activating mast cell chymases in mice (127).

Cathepsin W is expressed predominantly in NK cells and is up-regulated by IL-2, but not IL-12. NK cells contain, on average, 21 times more cathepsin W mRNA transcript than CTLs, whereas CD4^+^ T lymphocytes contain only traces. However, cathepsin W protein is not localized in secretory granules, like cathepsins C and H, but mainly in the endoplasmic reticulum, suggesting that it is not involved in pro-granzyme processing but rather plays a role in ER-resident proteolytic machinery (128).

Cysteine cathepsins have also been considered as an important element for protecting cytotoxic cells from their cytotoxic cargo (129). Thus, acidic conditions in cytotoxic granules inhibit granzyme activity and disable calcium binding to perforin, which is required for its activity (6). However, as cytotoxic cells are able to kill target cells *in vitro*, it is unclear how they are protected from the toxic effects of their granules, particularly perforin following its release into the cytolytic synapse. Balaji and collaborators suggested that cathepsin B might serve as a specific inhibitor of perforin following degranulation (129). However, CTLs from cathepsin B deficient mice were later shown to exhibit cytotoxic efficiency and survival on encounter with targets comparable to those of wild-type CTLs, questioning the role of cathepsin B in perforin inactivation (130).

Cathepsin K, expressed predominately in osteoclasts and involved in bone remodeling processes, also exists in immune cells, i.e., in cytoplasmic granules of multinucleated giant cells and in epithelioid cells, but not in normal resident macrophages (131). It has been implicated in the activation of dentritic cells and in contributing to autoimmune inflammatory processes (132).

## Regulation of Cell Cytotoxicity by Cysteine Cathepsins and Their Inhibitors

Predominant expression of particular cathepsins in cytotoxic cells and their subcellular localization, as well as their substrate specificity, make them likely candidates for the activation of cytotoxic zymogens. As noted earlier, perforin and granzymes require C- (perforin) or N- (granzymes) terminal processing in order to become activated and this activation was suggested to be accomplished by cysteine cathepsins. However, even though it has been shown *in vitro* that cathepsin L can process the C-terminal part of perforin and that its inhibition impairs cytotoxicity of human NK cell lines and mouse primary CTLs, *ex vivo* experiments on CTLs and NK cells from cathepsin L-deficient mice showed no difference in the effectiveness of target cell killing from that of controls (133), indicating that there are other proteases involved in this process. Cathepsin C has been proposed as the main protease to generate active granzymes from their precursor forms by proteolytic cleavage of the N-terminal dipeptide (134). However, the lymphocytes from cathepsin C-null mice express reduced, but still adequate, granzyme B activity and kill target cells almost as effectively as wild-type mice (135). In addition, lymphocytes derived from patients with Papillon–Lefèvre syndrome contain active granzyme B and kill target cells as effectively as healthy controls (125). Thus, at least for granzyme B, an alternative mechanism for processing and activation was proposed. Cathepsin H has been suggested as an alternative pro-granzyme B convertase; however, lymphocytes deficient in both cathepsin C and H are still able to generate active granzyme B, indicating that the involvement of other enzymes in pro-granzyme processing (42). Thus, based on current knowledge, functional redundancy in the activation of both granzymes and perforin could be an important adaptation in preventing tumor or pathogen-mediated immune suppression by inhibition of a single protease.

As in other cells, the activity of cysteine cathepsins in immune cells is controlled by endogenous cysteine protease inhibitors. Type I cystatin stefin A (also known as cystatin A) is present in follicular dendritic cells, in Hassall’s corpuscles and in thymic medullary cells, but not in lymphocytes (136). Macrophages contain stefin B (also known as cystatin B) and secrete it into the cell culture medium, while peripheral blood monocytes exhibit no stefin B immunoreactivity (137). The type II cystatins most abundant in immune cells are cystatin C and cystatin F (also known as leukocystatin and CST7). Cystatin C content was found to be higher in immature dendritic cells than in monocytes, promonocyte U-937 cells, and mature dendritic cells. The secretion of cystatin C from immature dendritic cells is in line with their strong endocytic activity and is decreased during maturation, in keeping with abolished endocytosis (109).

**Figure 2:**
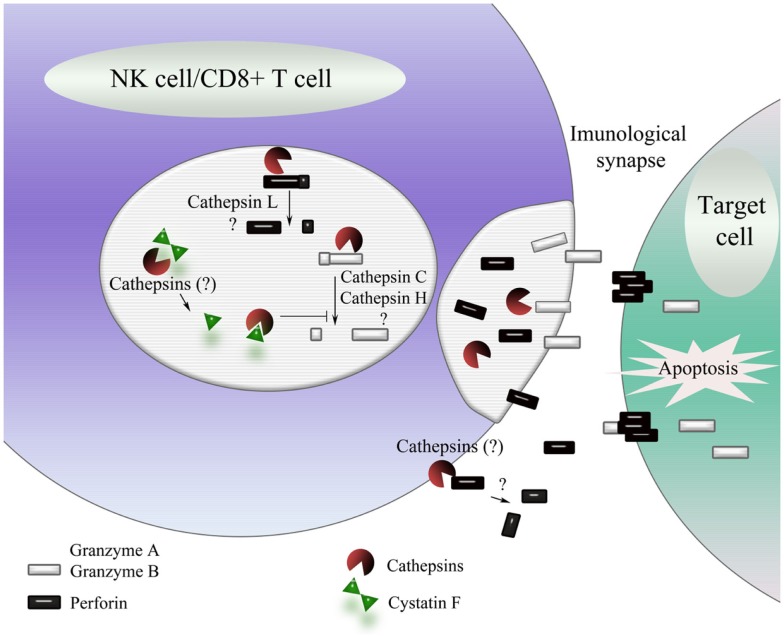
**Schematic representation of the regulation of NK cell/CD8^+^ T cell cytotoxicity by cathepsins**. On target cell recognition, cytotoxic cells secrete the content of their cytotoxic granules into the immunological synapse, leading to the caspase-dependent or independent cell death of target cells. The major cytotoxic mediators are activated by limited proteolysis by cathepsins C and H (granzyme A and B) and, possibly, cathepsin L (perforin). N-terminally processed cystatin F is a potent inhibitor of cathepsins C, H, and L and thus a potential regulator of NK cell/CD8^+^ T cell cytotoxicity. Exocytosed cysteine cathepsins may also be involved in inhibition of perforin, contributing to self-protection of cytotoxic cells from the contents of their cytotoxic granules.

Cystatin F is an exception among cystatins. After its synthesis, it is translocated to endosomes/lysosomes and is able to regulate cathepsin activity in these vesicles (138). It is predominantly expressed in lymphoid cells, with a preference for T cells, monocytes, NK cells, and dendritic cells. Cystatin F is produced in cells as a disulfide-linked dimer (139) inactive as an inhibitor of the C1 family of cysteine proteases (138). *In vitro*, unusually strong reducing conditions are needed to dissociate dimer to monomer. However, truncation of its N-terminal region, presumably by cathepsin V (also known as cathepsin L2/U) (140) (Figure 2), significantly enhances the monomerization and also changes the inhibitory properties of the resulting monomer (141). Intact monomeric cystatin F binds tightly to cysteine endopeptidases such as cathepsins L, F, K, and V, less tightly to cathepsins S and H, but not to exopeptidases cathepsins B, X, and C (138, 142, 143). Cystatin F, following N-terminal truncation, is a strong inhibitor of cathepsin C (141) but a weaker inhibitor of cathepsin S, whereas its ability to inhibit cathepsin H is only slightly increased (144). In cytotoxic cells, cystatin F therefore appears as a key regulator of granzyme processing and consequently cell cytotoxicity (Figure 2).

## Conclusion

Cysteine cathepsins are important players in the multiple processes of the immune response. Traditionally, these enzymes have been believed to execute proteolysis non-selectively within the lysosomes, to be redundant in their substrate specificity and to be expressed ubiquitously in cells and tissues. However, recently, this view has changed since restricted expression and specific enzymatic activity have been discovered for some of them. The role of cysteine cathepsins in CTLs and NK cells is particularly notable, since the ability of cathepsin C and cathepsin H to activate pro-granzymes in secretory granules is directly linked to the initiation of caspase-dependent or independent death of their target cells. Furthermore, cysteine cathepsins, like cathepsin L, can activate perforin, another key player in the cytotoxic process. Owing to their important role in cytotoxicity, the activity of cysteine cathepsins has to be precisely regulated. For that role, cystatin F, as the only endogenous cysteine protease inhibitor localized in endosomal/lysosomal vesicles, is a good candidate. Furthermore, since its activity is also regulated by proteolytic cleavage of its N-terminal peptide by, supposedly, cathepsin V, the latter may control the inhibitory activity of cystatin F and, hence, the rate of inhibition of pro-granzyme convertases cathepsins C and H. Owing to the specific roles of cathepsins C, H, and V and cystatin F in regulating cell cytotoxicity, they are potential therapeutic targets for improving cell therapy or impairing cytotoxicity in immune disorders. Future work therefore needs to be focused on understanding the fine-tuning in the cytotoxicity regulatory network, thus, enabling specific targets for immunotherapy to be identified.

## Conflict of Interest Statement

The authors declare that the research was conducted in the absence of any commercial or financial relationships that could be construed as a potential conflict of interest.
